# Incidence of malignancy after pediatric kidney transplantation: a single-center experience over the past three decades in Japan

**DOI:** 10.1007/s10157-021-02143-3

**Published:** 2021-09-27

**Authors:** Yujiro Aoki, Hiroyuki Satoh, Yuko Hamasaki, Riku Hamada, Ryoko Harada, Hiroshi Hataya, Kenji Ishikura, Masaki Muramatsu, Seiichiro Shishido, Ken Sakai

**Affiliations:** 1 Department of Urology, Tokyo Metropolitan Children’s Medical Center, Tokyo, Japan; 2 Department of Nephrology, Toho University Faculty of Medicine, 6-11-1 Omori-Nishi, Ota-ku, Tokyo, 143-8541 Japan; 3 Department of Nephrology, Tokyo Metropolitan Children’s Medical Center, Tokyo, Japan; 4 Department of General Pediatrics, Tokyo Metropolitan Children’s Medical Center, Tokyo, Japan; 5 Department of Pediatrics, Kitasato University School of Medicine, Kanagawa, Japan

**Keywords:** Kidney transplantation, Pediatric, Malignancy, Post-transplantation lymphoproliferative disease

## Abstract

**Background:**

Malignancy after kidney transplantation (KT) is one of the most serious post-transplant complications. This study aimed to investigate the incidence, type, and outcomes of malignancy after pediatric KT.

**Methods:**

We performed a retrospective cohort study on pediatric kidney transplant recipients aged 18 years or younger who received their first transplant between 1975 and 2009.

**Results:**

Among the 375 children who underwent KT, 212 were male (56.5%) and 163 were female (43.5%) (median age at KT, 9.6 years [interquartile range {IQR}] 5.8–12.9 years). The incidence of malignancy was 5.6% (*n* = 21). The cumulative incidences of cancer were 0.8%, 2.5%, 2.8%, 4.2%, 5.5%, and 15.6% at 1, 5, 10, 15, 20, and 30 years post-transplantation, respectively. Of 375 patients, 12 (3.2%) had solid cancer and nine (2.4%) had lymphoproliferative malignancy. The median age at the first malignancy was 21.3 years (IQR 11.5–33.3 years). The median times from transplant to diagnosis were 22.3 years (IQR 12.3–26.6 years) for solid cancer and 2.2 years (IQR 0.6–2.8) for lymphoproliferative malignancies. During follow-up, five recipients died due to malignancy. The causes of death were hepatocellular carcinoma in one patient, squamous cell carcinoma in the transplanted kidney in one patient, malignant schwannoma in one patient, and Epstein-Barr virus-related lymphoma in two patients. The mortality rate was 0.79 per 1000 person-years (95% confidence interval 0.38, 1.85).

**Conclusions:**

Early diagnosis and treatment of malignancies in transplant recipients is an important challenge. Therefore, enhanced surveillance and continued vigilance for malignancy following KT are necessary.

## Introduction

End-stage kidney failure is a rare and severe condition in children. Approximately 5–10 children per million in the age-related population start renal replacement therapy each year, and the mortality rate in children with end-stage kidney failure may be 30 times higher than that in the healthy age-related population [1, 2]. Pediatric kidney transplantation (KT) has become a standard renal replacement therapeutic option for chronic kidney disease (CKD), and the development and clinical application of new immunosuppressive agents has greatly improved KT outcomes, making it a well-established therapy [1]. Among the complications after KT, it is important to control cardiovascular diseases, infections, and malignancies that directly affect the prognosis of life. In this context, immunosuppressive therapy has been shown to increase the incidence of malignancy after KT [3].

Despite advances in immunosuppressants, patients who undergo pediatric KT have a five to ten times higher relative risk of cancer in the general population [4], and mortality due to malignancy after KT is ~ 11–18% [5, 6]. In particular, although calcineurin inhibitors, such as cyclosporin (CyA) and tacrolimus (Tac), have improved transplantation performance, post-transplant lymphoproliferative disease (PTLD) associated with Epstein–Barr virus (EBV) infection has increased [6]. Risk factors for PTLD include transplantation from an EBV-positive donor to an EBV-naive recipient, younger age at KT, and more aggressive immunosuppression. PTLDs are most likely to develop within the first year after transplantation [7, 8], and the incidence of PTLD in KT is reported to be 1.3% within 1 year and 2.4% within 5 years in children [9]. Furthermore, as post-transplant outcomes improve with long-term graft survival, indiscriminate and prolonged use of immunosuppressive drugs may not only cause various complications but also increase the risk of malignancy development; therefore, monitoring malignancies is an important issue.

The incidence and type of malignancy vary in different countries, and most reports are from Western countries. This study aimed to investigate the incidence, type, and outcomes of malignancies after pediatric KT at a single center in Japan.

## Materials and methods

### Ethics statements

This study was approved by the Research Ethics Committee (number H29b-91) before study commencement and complied with the Helsinki Declaration. The requirement for informed consent was waived by the committee.

### Study design and data collection

We performed a retrospective cohort study of consecutive pediatric patients who underwent primary KT from January 1975 to December 2009 at Tokyo Metropolitan Kiyose Children’s Hospital (predecessor of Tokyo Metropolitan Children’s Medical Center). During this period, 375 patients underwent primary KT at the age of ≤ 18 years. The patients were followed up from their KT to the last date that they were confirmed to be alive as of December 2016. Clinical data were collected from the medical records and included information on patient and donor characteristics, medical history, physical examination findings, immunosuppressive drugs used, type of malignancy, date of malignancy development, patient and graft survival, cause of death, and graft loss. The patients were divided into two groups based on the development of cancer, and the two groups were compared.

### Immunosuppression

The immunosuppressive protocol was divided into three periods. From 1975 to 1985 (era 1), the immunosuppressive protocol consisted of methylprednisolone (MPL), azathioprine (AZA), and/or mizoribine (MZ). In era 2, from 1986 to 2001, with the advent of CyA and Tac, the immunosuppressive protocol consisted of triple immunosuppression with MPL, AZA or MZ, and CyA or Tac. CyA and Tac were generally alternated in patients rather than randomized. Since 2002 (era 3), new immunosuppression regimens were introduced. Standard immunosuppression consisted of induction with basiliximab and triple therapy with MPL, CyA or Tac, and mycophenolate mofetil (MMF) [10]. CyA and Tac were generally alternated in patients rather than randomized. ABO-incompatible KT recipients underwent splenectomy at the time of KT, and rituximab was not used for B-cell depletion [11].

### Statistical analysis

To avoid potential selection bias, the study protocol was designed and approved before data collection began. Although the sample size was not predetermined, it was comparable to those commonly employed in similar studies. In addition, to avoid information bias, variables with missing values were excluded from the analysis.

All continuous data were checked for normality using the Shapiro–Wilk test. Categorical data are expressed as a number with a percentage, and continuous data are expressed as the mean ± standard deviation and median with a range or interquartile range (IQR), depending on the normality of the distribution. Categorical clinical variables were analyzed using the Pearson chi-square test or Fisher’s exact test. Continuous variables were analyzed using the Student *t *test, and qualitative variables were analyzed using the Mann–Whitney *U* test. Patient and graft survival were estimated using the Kaplan–Meier method and compared using the log-rank test and generalized Wilcoxon test. Factors associated with the incidence of malignancy were assessed using univariate and multivariate analyses according to a Cox proportional hazards model. Variables with a *P* value < 0.1 in the univariate analysis were included in the multivariate analysis. Multicollinearity between variables was evaluated using the variance inflation factor (VIF), with VIF > 10 signaling multicollinearity. The results are expressed as two-sided *P*-values, with *P* < 0.05 considered statistically significant. Statistical analyses were performed using SPSS software (version 26.0; IBM Corp., Armonk, NY, USA).

## Results

**Fig. 1:**
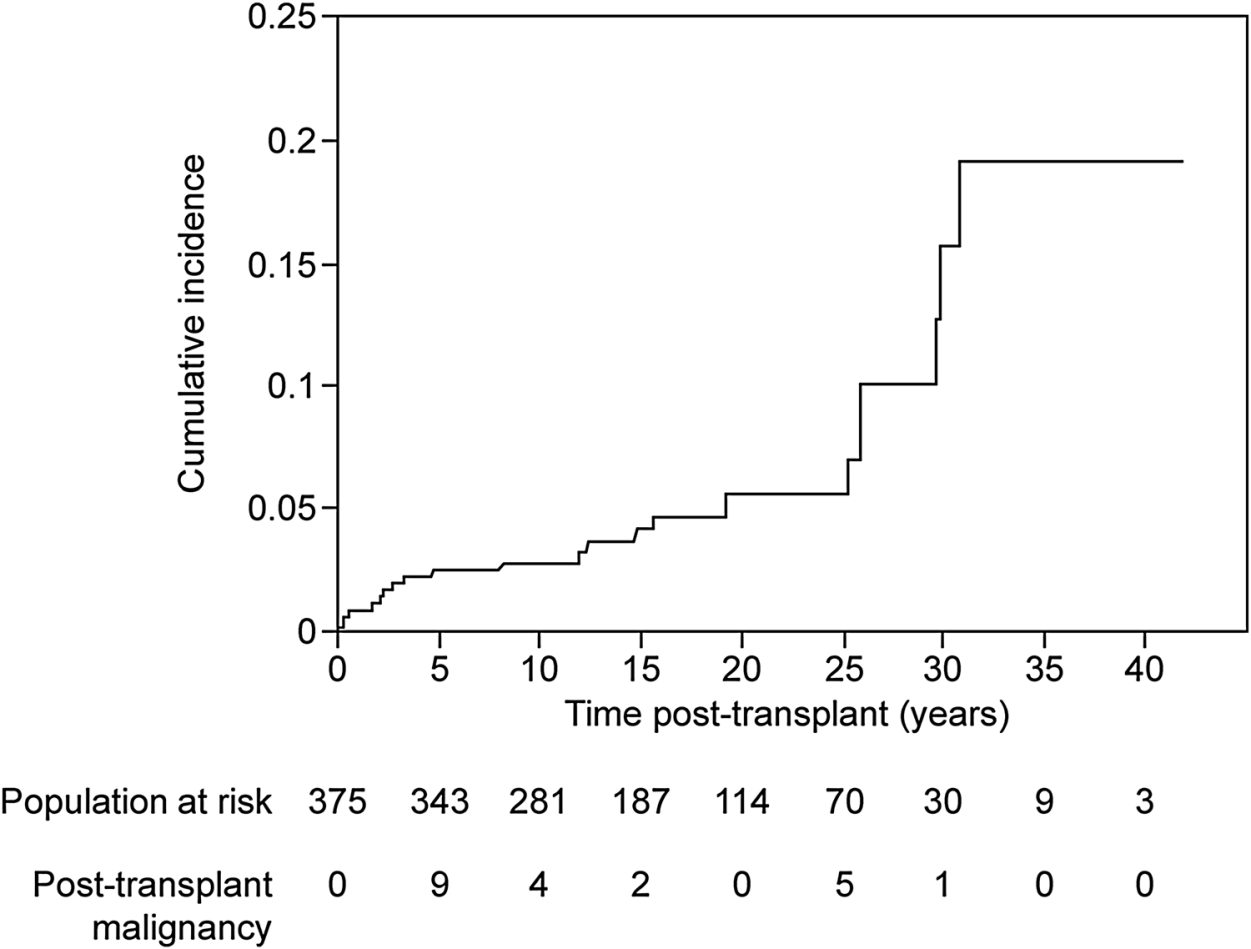
Estimated cumulative incidence of post-transplant malignancy after kidney transplant. Data shows the number of subjects at 0, 10, 20, 30, and 40 years post-transplant who were still at risk, and the cumulative number of subjects diagnosed with a PTM in our sample at those time points

During the follow-up period, 21 patients with post-transplant malignancy (PTM) were reported among 375 patients, with a crude rate of 5.6%. The cumulative incidences of PTM were 0.8% ± 0.5% at 1 year post-KT (360 patients at risk), 2.5% ± 0.8% at 5 years post-KT (343 patients at risk), 2.8% ± 0.9% at 10 years post-KT (281 patients at risk), 4.2% ± 1.2% at 15 years post-KT (187 patients at risk), 5.5% ± 1.5% at 20 years post-KT (114 patients at risk), and 15.6% ± 4.8% at 30 years post-KT (30 patients at risk) (Fig. 1). In the development of malignancy during the follow-up period, 6017.2 person-years were observed. The morbidity rate per 1000 person-years was 3.49 (95% confidence interval [CI] 2.28, 5.34).

The characteristics of the study population are summarized in Table 1. The cancer group showed a significant difference in recipient sex and donor type compared to the non-cancer group. There were no ABO-incompatible KTs in the cancer group. The median age at the first malignancy was 21.3 years (IQR 11.5–33.3 years). The median follow-up period was 15.2 years (IQR, 10.6–29.6 years). There were 12 patients (3.2%) with solid cancer and nine patients (2.4%) with PTLD during the observation period.

**Table 1:** Characteristics of the study population

Variable	Cancer	Non-cancer	*P*-value
	(*n* = 21)	(*n* = 354)	
Recipient age, years, median, [IQR]	11.3 [6.6–14.7]	9.5 [5.7–12.8]	0.15
Sex of recipient, male, *n* (%)	7 (33.3)	205 (57.9)	0.04
Number of re-transplants, *n* (%)	5 (23.8)	49 (13.8)	0.20
Preemptive KT, *n* (%)	2 (9.5)	27 (7.6)	0.67
Duration of dialysis, months, median, [IQR]	12.9 [6.4–26.9]	19.8 [10.3–40.7]	0.10
Donor age, years, median, [IQR]	38 [32–41]	39 [35–44]	0.16
Sex of donor, male, *n* (%)	5 (23.8)	138 (39.0)	0.25
Living related donor, *n* (%)	17 (81.0)	337 (95.2)	0.02
ABO-incompatible, *n* (%)	0 (0)	30 (8.5)	0.40
Primary immunosuppression, *n* (%)			
Cyclosporine	5 (23.8)	180 (50.8)	0.02
Tacrolimus	8 (38.1)	75 (21.2)	0.10
Azathioprine or mizoribine	13 (61.9)	256 (72.3)	0.46
Mycophenolate mofetil	8 (38.1)	98 (27.7)	0.32
Transplant era, *n* (%)			
Era 1 (1975–1985)	8 (38.1)	97 (27.4)	0.32
Era 2 (1986–2001)	5 (23.8)	149 (42.1)	0.11
Era 3 (2002–2009)	8 (38.1)	108 (30.5)	0.47
Acute rejection within 1 year after KT, *n* (%)	12 (57.1)	211 (59.6)	0.82
Graft loss, *n* (%)	14 (66.7)	146 (41.2)	0.04
Follow-up period, years, median, [IQR]	15.2 [10.6–29.6]	15.5 [10.3–22.4]	0.46

*IQR* interquartile range, *KT* kidney transplantation

Individual characteristics of patients with solid cancers are shown in Table 2. The median time from transplant to diagnosis of malignancy was 22.3 years (IQR 12.3–26.6 years) for solid cancer. In the case of Denys–Drash syndrome (case 12), upper mediastinal lymph node metastasis was observed at 4.8 years, and lung metastasis at 7.2 years after KT, both of which remitted with chemotherapy. This case started with a unilateral Wilms tumor, which metastasized to the lung at 10 months even after completion of chemotherapy for the primary disease. Three patients (25%) died of malignancy. The patient survival rate after treatment for solid cancer was 75% during the follow-up period.

**Table 2:** Characteristics of the individual patients with solid cancer

Case	Age at KT^a^ (years)	Sex	Primary disease	Duration of dialysis (months)	Number of KTs	Immunosuppression^a^	Type of malignancy	Time to diagnosis of cancer after KT (years)	Prognosis
1	16.5	M	CGN	5	1	MP, AZA	Liver cancer (HBV)	12.4	Dead
2	16.8	F	Hypo/dys	2	3	MP, AZA	Squamous cell carcinoma (transplanted kidney)	28.5	Dead
3	16.7	F	CGN	30	1	MP, AZA	Bladder cancer	26.0	Alive
4	7.1	F	FSGS	3	1	MP, AZA	Malignant schwannoma	25.3	Dead
5	5.5	M	Hypo/dys	7	1	MP, AZA	Liver cancer (HBV)	15.8	Alive
6	9.0	M	RPGN	15	2	MP, AZA	Testicular cancer	29.8	Alive
7	15.0	F	FSGS	13	1	MP, AZA	Bladder leiomyosarcoma	3.3	Alive
8	11.3	M	Hypo/dys	7	2	MP, MZ, AZA, ALG	Liver cancer (HCV)	29.9	Alive
9	14.0	F	FSGS	11	1	CyA, MP, AZA	Cervical cancer	26.0	Alive
10	14.0	F	RPGN	0	2	CyA, MP, AZA, ALG	Breast cancer	19.3	Alive
11	15.7	F	FSGS	16	1	Tac, MP, MMF, BLX	Breast cancer	12.0	Alive
12–1	14.2	F	Denys–Drash	9	1	CyA, MP, MMF, BLX	Wilms tumor (mediastinal lymph node metastasis)	4.8	Alive
12–2							Wilms tumor (lung metastases)	7.2	

*ALG* antilymphocyte globulin, *AZA* azathioprine, *BLX* basiliximab, *CGN* chronic glomerulonephritis, *CyA* cyclosporine, *F* female, *FSGS* focal segmental glomerulosclerosis, *Hypo/dys* hypoplastic/dysplastic kidney, *HBV* hepatitis B virus, *HCV* hepatitis C virus, *KT* kidney transplantation, *M* male, *MMF* mycophenolate mofetil, *MP* methylprednisolone, *MZ* mizoribine, *Tac* tacrolimus, *RPGN* rapidly progressive glomerulonephritis

^a^Primary kidney transplant

The characteristics of patients with PTLD are shown in Table 3. The median time from transplant to diagnosis was 2.2 years (IQR 0.6–2.8 years) for PTLD. All patients were treated with calcineurin inhibitors, and five patients (56%) were treated for acute rejection (AR) within 1 year after KT. Pre-transplantation EBV serology data in recipients and donors were not available in many cases. Pre-transplantation positive EBV serology was reported in 175 (46.7%) recipients and 188 (50.1%) donors. However, EBV serology status at the time of KT was unknown in 116 (30.9%) recipients and 181 (48.3%) donors. Even in eras 2 and 3 when calcineurin inhibitors were used, EBV serology status at the time of KT was unknown in 22 (8.2%) recipients and 76 (28.2%) donors. EBV serology at the time of KT in patients who developed PTLD was negative in six and positive in three. EBV serology of the donor was not available in three cases, while the combination of an EBV-negative recipient and an EBV-positive donor was discovered in five cases (56%). EBV serology of the recipient at the time of PTLD occurrence was positive in 60% of cases. Patient 2 underwent emergency surgery for gastrointestinal perforation but died of postoperative disseminated intravascular coagulation. Histopathology of the entire intestinal layer at the perforation site showed ulceration of the mucosal surface and cellular infiltration from the submucosa to the muscular layer, mainly of atypical lymphocytes, consistent with PTLD. Since EBV-DNA was identified in the tissue of the perforated small intestine, a diagnosis of gastrointestinal PTLD due to EBV infection was made. Despite treatment, two patients (22%) died due to PTLD. Graft loss occurred in case 1 2 years after PTLD treatment, and occurred in case 5 due to vascular injury during resection of the ileocecal tumor. The graft survival rate after treatment for PTLD was 44% during the follow-up period.

**Table 3:** Characteristics of individual patients with PTLD

Case	Age at KT^a^ (years)	Sex	Primary disease	Immunosuppression^a^	EBV serology at KT (recipient/donor)	EBV status of PTLD	Pathology/subtype	Time to diagnosis of cancer after KT	Symptoms	Localization	Treatment	Prognosis
1	15.6	M	MPGN	CyA, MP, AZA	P/NA	EBV negative	DLBCL	14.8 years	Abdominal pain	Colon, mesenteric lymph nodes	RI, surgery, chemotherapy	Alive
2	9.1	F	HUS	Tac, MP, MZ	N/P	EBV positive	Unclassifiable	4 months	Fever, upper abdominal pain	Gastroduodenal, small intestine	RI, antiviral drug, IVIG	Dead
3	2.8	M	Hypo/dys	Tac, MP, MZ	N/P	EBV positive	Early lesion	7 months	Vomiting, diarrhea, melena	Colon, mesenteric lymph nodes	RI, antiviral drug	Alive
4–1	9.8	F	Hypo/dys	Tac, MP, MMF	P/NA	EBV positive	Early lesion	1.8 years	Fever, vomiting, diarrhea	Colon, mesenteric lymph nodes	RI, antiviral drug, IVIG	Alive
4–2						EBV negative	Early lesion	8.3 years	Fever of unknown origin	Colon	RI, R, chemotherapy	
5	12.7	M	FSGS	Tac, MP, MMF, BLX	P/P	EBV negative	Burkitt lymphoma	8.1 years	Abdominal mass	Colon	RI, R, surgery, chemotherapy	Alive
6	11.0	F	Hypo/dys	CyA, MP, MMF, BLX	N/P	EBV positive	Early lesion	2.8 years	Upper abdominal pain	Colon, mesenteric lymph nodes	RI	Alive
7	4.3	F	Hypo/dys	Tac, MP, MMF, BLX	N/P	EBV positive	Monomorphic, T-/NK-cell	2.3 years	Fever, abdominal pain, diarrhea	Lymph nodes (neck, mediastinal, mesenteric)	RI, chemotherapy	Dead
8	3.8	M	Cortical necrosis	Tac, MP, MMF, BLX	N/NA	EBV positive	Polymorphic	3 months	Melena	Gastroduodenal, lymph nodes in porta hepatis	RI, R, chemotherapy	Alive
9	6.0	F	Hypo/dys	Tac, MP, MMF, BLX	N/P	EBV positive	Early lesion	2.2 years	Diarrhea, lymphadenopathy	Colon, lymph nodes (neck, inguinal)	RI, R	Alive

*AZA* azathioprine, *BLX* basiliximab, *CyA* cyclosporine, *DLBCL *diffuse large B-cell lymphoma, *EBV* Epstein-Barr virus, *F* female, *FSGS* focal segmental glomerulosclerosis, *HUS* hemolytic uremic syndrome, *Hypo/dys* hypoplastic/dysplastic kidney, *IVIG* intravenous immunoglobulin, *KT* kidney transplantation, *M* male, *MMF* mycophenolate mofetil, *MP* methylprednisolone, *MPGN* membranoproliferative glomerulonephritis, *MZ* mizoribine, *NA* not available, *N* negative, *P* positive, *PTLD* post-transplant lymphoproliferative disorders, *RI* reduction of immunosuppression, *R* rituximab, *Tac* tacrolimus

^a^Primary kidney transplant

The overall graft survival rates of the cancer and non-cancer groups were 95% and 91% at 1 year, 81% and 84% at 5 years, 56% and 75% at 10 years, 37% and 63% at 15 years, and 25% and 53% at 20 years, respectively. The graft survival rate of recipients with cancer was significantly lower than that of recipients without cancer (log-rank: *P* < 0.05, Wilcoxon: *P* < 0.01). The overall patient survival rates in the cancer group were 95%, 91%, 91%, 84%, and 84% at 1, 5, 10, 15, and 20 years after KT, respectively. Patient survival rates were not significantly different between the two groups. (log-rank: *P* = 0.19, Wilcoxon: *P* = 0.39).

After 6329.9 person-years of follow-up, the median follow-up duration was 15.4 years (IQR 10.3–22.5). Five patients died due to malignancy. The mortality rate per 1000 patient-years was 0.79 (95% CI 0.38, 1.85). The cause of death was hepatocellular carcinoma in one patient, squamous cell carcinoma in the transplanted kidney in one patient, malignant schwannoma in one patient, and EBV-related lymphoma in two patients. Of these, three patients died with a functioning graft. Nine patients had PTLD, with a median age of 9.8 years (IQR, 6.8–13.8 years). Two patients (one with gastrointestinal PTLD and one with T/NK-cell PTLD) died after diagnosis because of unresponsiveness to treatment. According to the Cox proportional hazards model, we assessed the variables related to the incidence of malignancy. Multivariate analysis identified era 3 versus era 1 (hazard ratio 2.20, 95% confidence interval 1.31–3.69) and era 3 versus era 2 (hazard ratio 2.27, 95% confidence interval 1.42–3.63) as independent risk factors for incidence of malignancy at 10 years post-transplantation (Table 4).

**Table 4:** Factors associated with incidence of malignancy at 10 years post-transplantation were analyzed using a Cox regression model

Variable	Univariate analysis				Multivariate analysis			
	HR	95% CI		*P*-value	HR	95% CI		*P*-value
Recipient age (years)	0.99	0.97	1.01	0.30				
Sex of recipient (male)	0.90	0.73	1.11	0.33				
Sex of donor (male)	0.98	0.79	1.21	0.86				
Living related donor	0.84	0.53	1.33	0.45				
Tac versus CyA	1.14	0.89	1.47	0.31				
MMF versus AZA or MZ	1.26	1.00	1.59	< 0.01				
Transplant era								
Era 3 versus Era 1	1.38	1.06	1.81	< 0.01	2.20	1.31	3.69	< 0.01
Era 3 versus Era 2	1.56	1.22	1.99	< 0.01	2.27	1.42	3.63	< 0.01
Graft loss	1.13	0.89	1.43	0.31				

*AZA* azathioprine, *CI* confidence interval, *CyA* cyclosporine, *HR* hazard ratio, *MMF* mycophenolate mofetil, *MZ* mizoribine, *Tac* tacrolimus

## Discussion

Malignancy after pediatric KT is a serious complication that affects morbidity and mortality. In this study, the overall incidence of PTM in our population was 5.6%, which is lower than that in patients in other countries [12–15]. The cumulative cancer incidence after pediatric KT has been reported to be 4–7% by 10 years [12, 15], 13–20% by 20 years [13–15], and 26–41% by 30 years [13, 15], which was higher than that in our study.

In contrast to adults, malignancies other than PTLD are rare in children. According to a report published by the North American Pediatric Renal Transplant Cooperative Study (NAPRTCS) [16], of the 12,189 pediatric patients who received transplants from 1987 to 2013, 311 (2.55%) developed post-transplant malignancies, of which 262 (84.5%) had lymphoproliferative diseases. As for malignancies other than lymphoproliferative diseases, skin cancer is most common, although cancers of various organs, sarcoma, melanoma, and neuroblastoma have been reported. Regarding the type of malignancy, the most common solid cancers in our series were hepatitis virus-related hepatocellular carcinoma and breast cancer; in contrast to other studies, there were no skin cancers. The type of solid cancer was very different from those reported in Europe, Austria, and the United States [11–14]. In our study, we also encountered sarcoma and liver cancer until the early 1980s. The reasons for this may include immunosuppression, mainly with steroids and metabolic antagonists, and the large number of hepatitis virus (HBV, HCV)-positive patients due to blood transfusion. The lower incidence of skin cancer may be at least partly explained by population and environmental differences in cancer risk. Furthermore, the incidence of childhood cancer in the Japanese population is lower than that in other countries, e.g., England; this is thought to be due to racial differences and genetic factors [17]. Therefore, it is possible that the incidence of solid cancers after pediatric KT may also be lower in Japan than in other countries.

The median time to cancer development after pediatric KT varied by cancer type. PTLD developed early after transplantation, while solid tumors developed mostly after the transition to adulthood. Francis et al. [14] reported two peaks in the time to cancer for non-skin cancers, with a median of 6.6 years for PTLD and a median of 14.8 years for other cancers, which are similar to the findings in our study. This suggests that surveillance for PTM associated with pediatric KT should focus on PTLD during the first decade after KT, and that adult-type cancers should be included in the differential diagnosis thereafter.

Recent reports have shown that transplant recipients have a higher risk of death from cancer than do other patients with cancer [18]. In a study of pediatric solid organ transplant recipients, 23% of deaths were due to malignancy, and 68% of cancers causing death were PTLD [19]. Additionally, a study of pediatric kidney transplant recipients reported that 64% of deaths were due to cancer [15]. The impact of PTM on the graft has been debated, and no definitive opinion has been reached. Serrano et al. [15] reported that the hazard ratios for both death and graft loss were higher in patients with PTM than in those without PTM, whereas Francis et al. [20] reported that the development of PTM was associated only with death and not with graft loss. In our study, four of the patients with PTM died with a functioning graft, and one surviving patient progressed to kidney failure because of chronic allograft nephropathy after PTM treatment. The Kaplan–Meier curve showed a significant difference in graft survival of patients with PTM compared to patients without PTM, but no significant difference in patient survival. In our study, we were also unable to demonstrate the impact of PTM development on allografts.

PTLD is the most common malignancy observed after pediatric KT. The incidence of PTLD is higher in pediatric KT recipients than in adult KT recipients, and is seen in 2–4% of pediatric KT patients at a median of 14.9 months from transplant [7]. The major risk factors for the development of PTLD are the degree of T-cell immunosuppression and the EBV serological status of the recipient [7–9]. PTLD is an EBV-positive B-cell proliferation that occurs in immunosuppressed patients and reduces T-cell immune surveillance. Because EBV-infected B cells are normally suppressed by cytotoxic T-cells, PTLD may develop when T-cell immunity is compromised. The relationship between immunosuppression after pediatric KT and the risk of developing PTLD has been shown to increase with an increase in the degree of immunosuppression in patients receiving induction therapy or long-term high-dose Tac [21–23]. In our study, we investigated risk factors for the incidence of malignancy up to 10 years after KT and identified era3 using Tac and MMF as independent risk factors. Therefore, it was suggested that the recent immunosuppressive therapy has an impact on the incidence of malignancy (especially on the incidence of PTLD). In the present study, most pediatric KT patients who developed PTLD were treated with Tac and MMF. In addition, more than 50% of patients were treated for AR within 1 year after KT, suggesting that they were in a state of over-immunosuppression. The highest-risk group comprises EBV-naïve recipients transplanted with kidneys from EBV-positive donors, and the cause of PTLD in more than 90% of pediatric cases is the proliferation of EBV-positive B cells [18]. In our study, EBV serology of the recipient at the time of PTLD occurrence was positive in 60% of cases, but we could not investigate further because little donor serological information was available. In previous reports, donor positivity/recipient negativity (hazard ratio 7.7, 95% CI 1.6–35.9) was a risk factor for PTLD in EBV serotypes [21]. Therefore, pediatric patients with KT are generally considered to be at a higher risk of developing PTLD because of the higher pre-transplant EBV seronegative rate in children.

Recently, regular monitoring of EBV viral load and early recognition of recipients at high risk for PTLD has been considered a clinical priority [24]. Previous studies have shown that elevated EBV-DNA levels and persistently high EBV load are risk factors for PTLD [25, 26], but no clear cut-off EBV load for prediction of the development of PTLD has been determined. However, EBV-DNA levels should be monitored regularly in patients at high risk for PTLD, as they indicate EBV infection and/or reactivation, detected as an increase in EBV-DNA in the peripheral blood preceding PTLD. Regarding monitoring intervals, since most cases of EBV-positive PTLD develop early after KT, it is reasonable to monitor high-risk patients frequently in the early post-KT period and to increase the monitoring interval as the time after KT increases. The decision to reduce the frequency of EBV monitoring after KT must be made on an individual basis, considering many factors, including the type of graft, degree of ongoing immunosuppression, and EBV viral load.

The present study has numerous limitations due to the retrospective nature of the study and its single-center design. First, our study had a small sample size, and the heterogeneity of our cohort potentially limits the generalizability of our findings. Second, maintenance therapy for immunosuppression has changed over time, and conclusions about long-term cancer risk cannot be drawn solely from protocols for induction therapy for immunosuppression. In addition, there were several cases of unknown EBV serology among the recipients and donors, and the relationship between EBV serology and the development of PTLD could not be shown. Finally, the management and surveillance of transplant immunity and oncology has changed over a short period, and given the long duration of the study, there are limitations to this study that are influenced by time. The best way to address this issue would be to perform a multicenter, prospective study rather than a single-center, retrospective study.

## Conclusions

In this study, we reported the long-term results of the incidence of malignancy after pediatric KT in Japanese patients at a single center. KT is the treatment of choice for children with CKD because it provides the best opportunities for growth, development, and quality of life. In recent years, the results of pediatric KT have improved dramatically owing to improvements in perioperative and postoperative care, immunosuppressive medications, and infection surveillance and management. However, malignancy after KT is a serious post-transplant complication, and the long-term risk of malignancy increases significantly after KT. Therefore, the occurrence of malignancy after pediatric renal transplantation is an important factor in mortality. Regular surveillance after KT should be strengthened, and continued vigilance for detection of malignancy following KT is necessary.
